# Initial Experience With 6D Skull Tracking and Intrafractional Motion Monitoring in the United Arab Emirates' First CyberKnife® Radiosurgery Center

**DOI:** 10.7759/cureus.52143

**Published:** 2024-01-11

**Authors:** Teekendra Singh, Dimpi Singh, Sinead Catherine Murphy, Abdulrahman Bin Sumaida, Nandan M Shanbhag

**Affiliations:** 1 Oncology and Radiosurgery, Neuro Spinal Hospital, Dubai, ARE; 2 Health Informatics, Mahatma Gandhi Institute of Health Informatics, Jaipur, IND; 3 Oncology/Radiotherapy, Neuro Spinal Hospital, Dubai, ARE; 4 Oncology/Radiation Oncology, Tawam Hospital, Al Ain, ARE; 5 Oncology, Tawam Hospital, Al Ain, ARE; 6 Internal Medicine, College of Medicine and Health Sciences, United Arab Emirates University, Al Ain, ARE

**Keywords:** cyberknife® radiosurgery, intrafractional motion monitoring, 6d-skull tracking, stereotactic radiotherapy, patient movement analysis, treatment precision, adaptive radiosurgery, real-time tracking technology

## Abstract

Introduction

The introduction of the CyberKnife® system has marked a significant advancement in the field of radiosurgery, offering unparalleled precision in targeting and treating cranial and extracranial lesions. This paper details the first experience from the United Arab Emirates in implementing 6D skull tracking and intrafractional motion monitoring in CyberKnife® radiosurgery. The study aims to evaluate the system's efficacy in tracking and adjusting patient movement during treatment, enhancing treatment accuracy and patient safety.

Methods and materials

This retrospective study analyzed 732 images from six patients treated at the UAE's first CyberKnife® center. Patients were divided into two groups based on their treatment regimens: Patients 1 to 4 (P1 to P4) received multifractionated stereotactic radiotherapy, while Patients 5 and 6 (P5 and P6) underwent single-fraction stereotactic radiosurgery (SRS). The movements recorded included supero-inferior, lateral, antero-posterior, roll, pitch, and yaw. Statistical tools were employed to interpret the data, including heat maps, box-and-whisker plots, and correlation analysis.

Results

The study's results indicate varied patterns of intrafractional movement across the different axes and between the two treatment groups. Multifractionated therapy patients exhibited a specific range and frequency of movements compared to those undergoing single-fraction treatment. The most significant movements were observed in the supero-inferior and lateral axes.

Discussion

The findings suggest that the CyberKnife® system's real-time tracking and adaptive capabilities are crucial in managing patient movements, especially in prolonged treatment sessions. The differences in movement patterns between multifractionated and single-fraction treatments underscore the need for tailored approaches in intrafractional motion monitoring.

Conclusion

The initial experience of the UAE's first CyberKnife® center demonstrates the system's effectiveness in addressing intrafractional movements, enhancing the precision and safety of radiosurgery treatments. This study contributes valuable insights into optimizing treatment protocols and underscores the importance of continuous monitoring and adaptive strategies in advanced radiosurgery.

## Introduction

The CyberKnife® system represents a significant breakthrough in radiosurgery, particularly for its precise targeting of cranial and extracranial lesions. This advanced technology has been a game-changer in treating various tumors, enhancing the accuracy and safety of radiological procedures. Its introduction in the United Arab Emirates, with the innovative use of 6D skull tracking and intrafractional motion monitoring, marks a significant step forward in the global application of this technology.

The efficacy of the CyberKnife® system in treating intracranial tumors has been well documented, with studies showing its success in treating brain and spinal cord tumors, which has paved the way for its use in extracranial sites [1]. This versatility is further emphasized by research demonstrating a high local control rate of 96% in treating benign extracranial tumors, showcasing the system's effectiveness and safety [2]. Additionally, the tolerance of CyberKnife® radiosurgery for intracranial and extracranial tumors in the skull base has been highlighted, indicating its broad scope of application [3].

Moreover, the CyberKnife® system has shown significant potential in treating large, benign tumors in delicate areas such as the brain and cranial base. Studies have found that multisession hypofractionated CyberKnife® radiosurgery (hSRS) is an effective and safe option for these complex lesions [4]. The system's capacity to deliver precise, stereotactic radiation to the spine and extracranial targets, which enhances patient comfort during treatment, has been noted [5]. Its adaptability is further demonstrated in its potential for treating various extracranial sites and its effectiveness in spinal lesion treatment without causing acute radiation toxicity or new neurological deficits [6,7].

The practical applications of the CyberKnife® system have extended to treating lesions in the thorax and abdomen, demonstrating its versatility beyond just cranial applications [8]. Its ability to treat tumors outside the intracranial compartment, including those that move with respiration, has showcased the system's adaptability [9]. Furthermore, CyberKnife® radiosurgery's effectiveness and low toxicity in treating recurrent brain metastases have been established, underscoring its repeatability and reliability in such treatments [10].

In summary, introducing the CyberKnife® system in the UAE, equipped with advanced features like 6D skull tracking and intrafractional motion monitoring, exemplifies the system's role in enhancing treatment precision and patient safety in cranial and extracranial cases. The system has established itself as a highly effective and versatile tool in the field of radiosurgery, offering new hope and possibilities in treating various tumors.

## Materials and methods

This retrospective analysis was conducted at the United Arab Emirates inaugural CyberKnife® center. The study aimed to evaluate the precision of intrafractional motion monitoring facilitated by the CyberKnife® system, specifically focusing on its 6D skull-tracking capability.

Six patients undergoing radiosurgery were selected for this study between November 2021 and February 2022. The inclusion criteria included patients receiving hypofractionated stereotactic radiosurgery (hSRS) or single-fraction stereotactic radiosurgery (SRS). Patients P1 to P4 were treated with hSRS, while P5 and P6 underwent SRS. The exclusion criteria included patients with incomplete treatment data or those who underwent treatment for non-cranial lesions.

Tracking technology and data acquisition

All participants in the study were positioned in a stable, supine head-first posture for treatment, utilizing a tailor-made pillow (AccuForm™, Civco Medical Systems, Orange City, FL), molded to conform to the head's base and designed to maintain its form indefinitely. A thin, non-invasive thermoplastic mask, approximately 2.4 mm thick, was shaped to fit each patient's face and secured to an acrylic frame attached to the CyberKnife® robotic couch. The advanced image guidance capabilities of the CyberKnife® system eliminated the need for external fiducial markers during setup and throughout the treatment process.

A detailed CT scan of the head was conducted using a non-contrast, helical technique, producing axial slices with a 1 mm thickness. Additionally, a contrast-enhanced T1-weighted MRI scan was obtained and subsequently merged with the CT images for enhanced delineation of the tumor and surrounding normal tissues.

Image fusion, target outlining, and treatment planning were done using the Multiplan® software (Accuray Inc., Sunnyvale, CA). A radiation oncologist meticulously contoured the clinical target volume (CTV) and adjacent organs at risk to maintain dosage limits. In all instances, the planning target volume (PTV) was set to match the CTV, with tumor targeting refined exclusively by selecting appropriate isodose levels. The ideal treatment plan aimed to administer SRS/hSRS at an isodose line of 80-85%, covering 100% of the PTV's prescribed dose. Treatment plans were evaluated based on target coverage, dose heterogeneity, and the conformality index. The Multiplan® system generated multiple series of digitally reconstructed radiographs (DRRs) from the CT data, which were utilized to align the patient accurately during treatment sessions.

In the CyberKnife® treatment process, patients were positioned on the robotic treatment couch, replicating their simulation position with in-room laser guidance. The system employed the 6D skull-tracking mode, leveraging the skull's structural features to monitor and adjust for movements in all six directions. This mode established a fixed relationship between the target volume and the skull's skeletal characteristics.

For initial alignment, the treatment protocol involved acquiring orthogonal kilovoltage X-ray images with the in-room Target Locating System (TLS), which were then cross-referenced with the treatment planning system (TPS)-generated DRRs. Any discrepancies in positioning were automatically rectified by adjusting the robotic couch, ensuring precise alignment with the planned treatment position.

The treatment begins with a pre-set default interval for image acquisition. After confirming the stability of the target in the initial phase of treatment, the imaging interval is dynamically adjusted between 5 and 150 seconds based on the observed movements of the target. Throughout the session, the TLS continuously compares live images against a series of DRRs, providing detailed data on six-dimensional intrafraction movements. Once treatment commenced, the system continuously monitored and corrected real-time setup errors using the robotic manipulator. The CyberKnife® robot can autonomously correct deviations up to ±10 mm in translational axes and ±1.5 degrees in rotational axes, maintaining clinical precision within <0.5 mm.

Seven hundred and thirty-two images were acquired during treatment sessions using the CyberKnife® system's imaging and tracking technology. These images were analyzed to record movements across six axes: supero-inferior (S-I), lateral, antero-posterior (A-P), roll, pitch, and yaw.

Data analysis

Statistical analyses were performed on the collected data to evaluate the patterns and magnitude of intrafractional movements. Tools such as heatmaps, box-and-whisker plots, and correlation matrices were utilized to interpret the movement data. The analysis compared movement patterns between patients undergoing hSRS and those receiving SRS.

Ethical considerations

All procedures followed were per the international ethical standards, and informed consent was obtained from all patients included in the study.

## Results

Patients' diagnoses and characteristics are summarized in Table 1.

**Table 1:** Patient characteristics

Tumor type	Number of cases	Gender	Age in years
Acoustic neuroma	2	1 Male; 1 female	35; 30
Meningioma	1	1 Female	23
Tectal glioma	1	1 Male	37
Vestibular schwannoma	1	1 Male	64
Temporal cavernoma	1	1 Male	47
Cases/Gender (total)	Cases	Male/Female	
	6	4/2	
Age in years	Min	Max	Median
	23	64	36

The treatment parameters used for individual patients are summarized in Table 2.

**Table 2:** Treatment parameters MU, monitor units.

Patient no.	Prescribed dose	Total number of beams	Total MU	Treatment time (min)	Collimator (mm)
1	25 Gy in 5 fractions	93	32,189	22	Fixed 7.5
2	21 Gy in 3 fractions	125	27,252	29	Fixed 7.5
3	25 Gy in 5 fractions	99	37,819	29	Fixed 7.5
4	25 Gy in 5 fractions	127	22,264	24	Fixed 7.5
5	16 Gy in 1 fraction	101	7,254	34	Fixed 7.5
6	14 Gy in 1 fraction	115	17,823	34	Fixed 7.5

**Figure 1:**
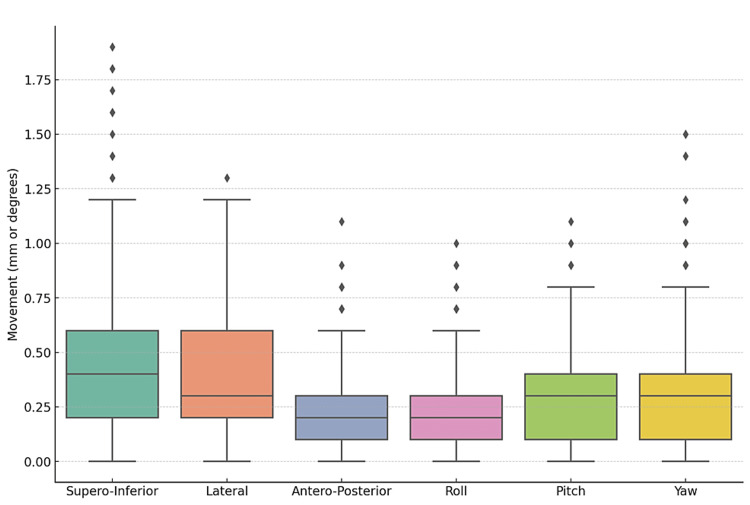
Box-and-whisker plot of movements The figure depicts minimum, median, and maximum shifts in each of the directions during patient treatment.

Before treatment on CyberKnife®, a patient-specific quality assurance (QA) was performed for each patient to ensure smooth execution of the treatment plan. The mean intrafraction translational setup errors were 0.45 mm (SD ±0.32) in the S-I direction, 0.37 mm (SD ±0.26 mm) in the lateral direction, and 0.21 mm (SD ±0.16 mm) in the A-P direction. In the overall pooled dataset, 93.57% (685/732) shifts in the S-I direction, 98.36% (720/732) shifts in the lateral direction, and 100% (732/732) shifts in the A-P direction were <1 mm and the maximum shifts were encountered as 1.9 mm, 1.3 mm, and 1 mm in S-I, lateral, and A-P directions, respectively (Figure 1).

Mean intrafraction rotational setup errors were 0.21 degree (SD ± 0.19 degree) roll, 0.30 degree (SD ± 0.22 degree) pitch, and 0.31 degree (SD ± 0.25 degree) yaw. 100% (732/732) shifts in pitch direction, 99.59% (729/732) shifts in roll direction, and 96.99% (710/732) shifts in yaw direction were <1 degree, and the maximum shifts were encountered as 1 degree, 1.1 degrees, and 1.5 degree in roll, pitch, and yaw directions, respectively. None of the instances encountered during any of the fractions where the tolerance of the robotic correction threshold was exceeded requires treatment interruption or manual couch correction. The mean 3D vector displacement for overall translational shifts was 0.38 mm (Table 3).

**Table 3:** Three-dimensional vector displacement of individual patients in translation directions S-I, supero-inferior; A-P, antero-posterior; P, patient.

	P1	P2	P3	P4	P5	P6
S-I (mm)	0.43	0.48	0.63	0.70	0.48	0.33
Lateral (mm)	0.44	0.44	0.52	0.49	0.17	0.30
A-P (mm)	0.31	0.26	0.24	0.28	0.19	0.19
Mean (mm)	0.39	0.39	0.46	0.49	0.28	0.27

**Figure 2:**
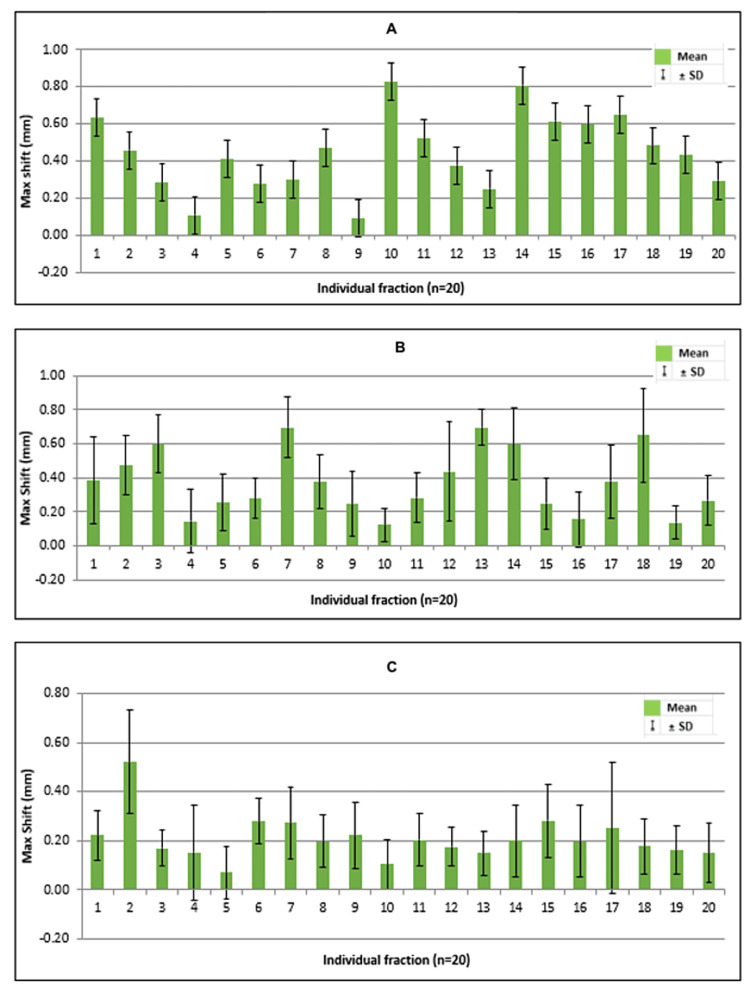
Movements for each fraction A: superoinferior; B: lateral; C: anteroposterior; the figure depicts shifts in millimeters (mm) in the primary directions for each fraction; total fractions were 20.

**Figure 3:**
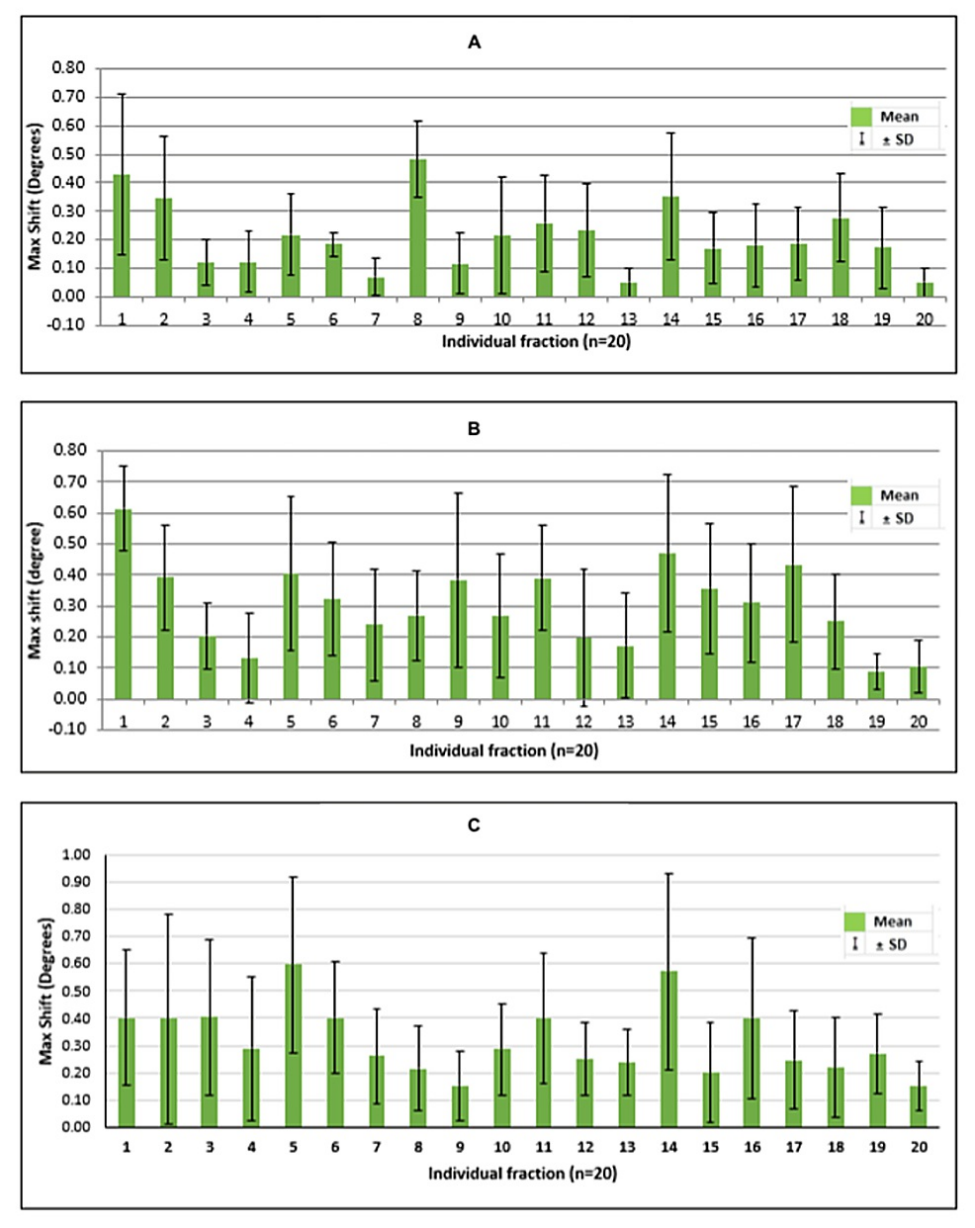
Shifts for each fraction A: roll; B: pitch; C: yaw; the figure depicts the shifts in degrees in roll, pitch, and yaw for each fraction; the total fractions were 20.

The individual mean intrafraction errors for each fraction (n = 20) showing the range of shifts in translational and rotational directions have been shown in the images (Figures 2A-2C, 3A-3C).

The calculated systemic error was 0.22 mm, 0.17 mm, and 0.13 mm, and the random error was 0.26 mm, 0.18 mm, and 0.14 mm in S-I, lateral, and A-P directions, respectively. Using the Van-Herk formula, the calculated PTV margins were 0.7 mm, 0.5 mm, and 0.4 mm in S-I, lateral, and A-P directions, respectively. 

**Figure 4:**
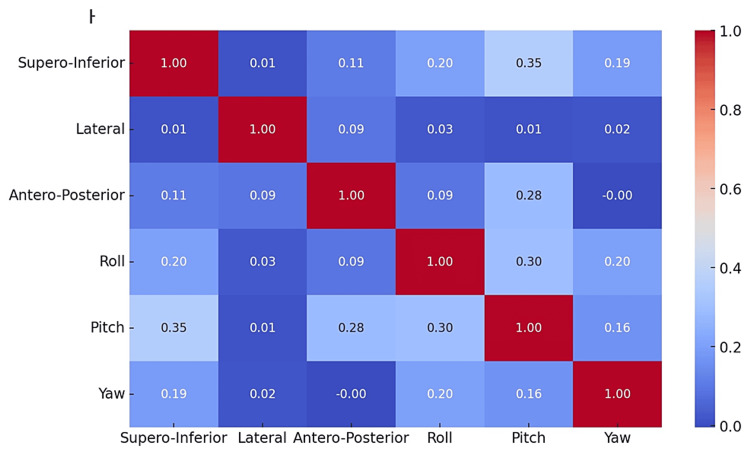
Heatmap showing the correlation between different movements

The correlation matrix table shows the correlation coefficients between different types of movements. Values closer to 1 or -1 indicate stronger positive or negative correlations, respectively, while values near 0 suggest a lack of correlation (Figure 4).

**Figure 5:**
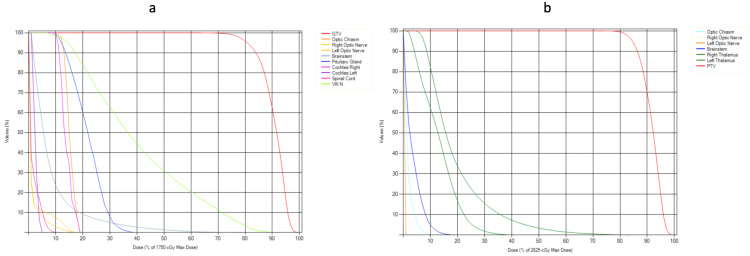
Dose-volume histogram The figure depicts the relative dose to the target organ and the organs at risk in a stereotactic radiosurgery (SRS) patient (a) and a hypofractionated patient treated with three fractions (b). GTV, gross tumor volume; PTV, planning target volume.

The dose-volume histogram for an SRS and hypofractionated SRS is depicted in Figure 5. 

## Discussion

The emergence of CyberKnife® as a groundbreaking technology in treating intracranial tumors marks a significant advancement in radiosurgery. This system has been pivotal in providing highly precise and effective treatment options for complex intracranial conditions, offering new hope to patients with inoperable or surgically complex tumors [11]. The CyberKnife's ability to treat tumors outside the intracranial compartment, including those that move with respiration, demonstrates its adaptability and precision, thereby widening the scope of its applications [12].

The CyberKnife® system's state-of-the-art technology has shown impressive tumor control rates exceeding 90% for various intracranial tumors such as pituitary, acoustic, and meningiomas [13]. These high success rates underscore the system's efficacy in managing complex intracranial conditions. The technology has also been effectively used to treat other intracranial lesions like meningiomas, acoustic schwannomas, brain oligometastases, and skull base tumors like chordomas, proving its utility across a range of intracranial conditions [14].

The CyberKnife® system offers a significant advantage over traditional radiosurgery techniques, particularly for treating metastatic brain tumors. Studies have demonstrated excellent local control with acceptable toxicity levels in patients with melanoma or renal cell brain metastases, further attesting to its safety and effectiveness [15]. Moreover, the frameless nature of the CyberKnife® system, along with its advanced imaging capabilities, provides extreme accuracy and comfort during treatment [16].

The CyberKnife® has been instrumental in treating multiple brain metastases, offering high tumor control with low toxicity and allowing for the retreatment of recurrent metastases [17]. Effectively managing recurrent conditions is crucial in improving patient outcomes and quality of life. Additionally, CyberKnife's comparable dose fall-off characteristics to other radiosurgical systems like Gamma Knife and Novalis systems favor hypofractionated treatments for fast-growing tumors, providing a more tailored approach to treatment [18].

The results from the study using the Cyberknife® M6 robotic radiosurgery system at our center in the United Arab Emirates are noteworthy, particularly in the context of being first in the region for treating brain tumors with frameless stereotactic radiosurgery/radiotherapy (SRS/SRT) [19,20]. These results have significant implications for treating intracranial tumors, especially regarding precision, safety, and treatment margins.

The finding that mean intrafraction translational setup errors are minimal (0.45 mm in the S-I direction, 0.37 mm in the lateral direction, and 0.21 mm in the A-P direction) is particularly impressive [21]. This high level of precision in positioning aligns with other studies highlighting the CyberKnife system's sub-millimeter accuracy, which is crucial for effective and safe radiological interventions [22]. Such precision is critical when dealing with sensitive intracranial structures, where minor deviations can have significant consequences.

The reported rotational setup errors (0.21-degree roll, 0.30-degree pitch, and 0.31-degree yaw) and the fact that most shifts in all directions were less than 1 mm or 1 degree demonstrate the system's reliability and stability during treatment [23]. These findings are critical, considering the delicate nature of intracranial structures and the need for precise targeting to avoid damaging healthy brain tissue.

The absence of instances requiring treatment interruption or manual couch correction due to exceeding the robotic correction threshold speaks to CyberKnife's real-time monitoring and adjustment efficacy. This capability is essential for maintaining treatment accuracy throughout the procedure, especially for lesions prone to movement, such as those influenced by respiration [24].

The calculated PTV margins based on the Van-Herk formula (0.7 mm in S-I, 0.5 mm in lateral, and 0.4 mm in A-P directions) suggest the CyberKnife® system's highly precise targeting ability. These narrow margins indicate the system's high-level accuracy, reducing the risk of irradiating surrounding healthy tissues [25]. This precision is particularly beneficial in treating brain tumors, where the goal is to maximize tumor control while minimizing potential damage to adjacent critical structures.

Limitations of the study

First, the study may be limited by its sample size and diversity; if the patient cohort was small or not representative of the broader population regarding tumor types and locations, the results might lack generalizability. Secondly, the absence of long-term follow-up data restricts insights into the persistence of treatment effects, late-onset side effects, and long-term tumor control. Thirdly, the study might have overlooked patient-reported outcomes, such as quality of life and treatment experience, which are crucial for a holistic understanding of the treatment's impact.

## Conclusions

In conclusion, the study conducted using the CyberKnife® M6 robotic radiosurgery system offers valuable insights into the advanced treatment of intracranial tumors. The results demonstrate the system's remarkable precision and reliability, with minimal intrafractional motion and highly accurate targeting capabilities. These findings emphasize the potential of the CyberKnife® system in enhancing the safety and efficacy of radiological treatments for brain tumors, especially in cases where traditional surgical approaches are not feasible or carry significant risks. The study also highlights the system's ability to maintain accuracy throughout treatment, ensuring minimal impact on the surrounding healthy tissues.

However, it is essential to consider the study's limitations, including the need for larger and more diverse patient samples, longer-term follow-up data, comparative analyses with other treatment modalities, and a more comprehensive inclusion of patient-reported outcomes. Future research addressing these areas will be instrumental in further establishing the role of the CyberKnife® system in the evolving landscape of intracranial tumor treatment, ultimately contributing to improved patient outcomes in the United Arab Emirates and beyond.
